# A Compend of Gynæcology

**Published:** 1891-05

**Authors:** 


A COMPEND OF GYNÆCOLOGY. By Henry Morris, M. D. P. Blackiston, Son & Co. Philadelphia. 1891.

While this is No. 7 of the Query-Compend series, and is intended mainly for medical students, the book is of such merit as to deserve a place in the library of every practical physician· The author handles the subject with clearness and precision, while his conservatism in the use of instruments is especially commendable. W. A. C.

